# Biases in the spectral amplitude distribution of a natural scene modulate horizontal size perception

**DOI:** 10.3389/fpsyg.2023.1247687

**Published:** 2023-12-06

**Authors:** Pablo Sanz Diez, Sandra Gisbert, Annalisa Bosco, Patrizia Fattori, Siegfried Wahl

**Affiliations:** ^1^Institute for Ophthalmic Research, Eberhard Karls University Tuebingen, Tuebingen, Germany; ^2^Carl Zeiss Vision International GmbH, Aalen, Germany; ^3^Department of Biomedical and Neuromotor Sciences, University of Bologna, Bologna, Italy; ^4^Alma Mater Research Institute for Human-Centered Artificial Intelligence (Alma Human AI), University of Bologna, Bologna, Italy

**Keywords:** size perception, visual adaptation, adaptation aftereffects, spatial frequency, spatial distortion, spatial distribution, manual estimate aperture

## Abstract

**Introduction:**

Visual perception is a complex process that involves the analysis of different spatial and temporal features of the visual environment. One critical aspect of this process is adaptation, which allows the visual system to adjust its sensitivity to specific features based on the context of the environment. Numerous theories highlight the significance of the visual scene and its spectral properties in perceptual and adaptation mechanisms. For example, size perception is known to be influenced by the spatial frequency content of the visual scene. Nonetheless, several inquiries still exist, including how specific spectral properties of the scene play a role in size perception and adaptation mechanisms.

**Methods:**

In this study, we explore aftereffects on size perception following adaptation to a natural scene with a biased spectral amplitude distribution. Twenty participants had to manually estimate the horizontal size of a projected rectangle after adaptation to three visually biased conditions: vertical-biased, non-biased, and horizontal-biased. Size adaptation aftereffects were quantified by comparing the perceptual responses from the non-biased condition with the vertical- and horizontal-biased conditions.

**Results:**

We found size perception shifts which were contingent upon the specific orientation and spatial frequency distribution inherent in the amplitude spectra of the adaptation stimuli. Particularly, adaptation to vertical-biased produced a horizontal enlargement, while adaptation to horizontal-biased generated a decrease in the horizontal size perception of the rectangle. On average, size perception was modulated by 5–6%.

**Discussion:**

These findings provide supporting evidence for the hypothesis that the neural mechanisms responsible for processing spatial frequency channels are involved in the encoding and perception of size information. The implications for neural mechanisms underlying spatial frequency and size information encoding are discussed.

## 1 Introduction

Perception is an area that consistently stimulates substantial scientific interest. The concept of human perception involves a broad spectrum of sensory modalities, among which the visual modality holds a pivotal role from both a scientific and societal perspective (Hutmacher, 2019). Visual perception entails the coordination and integration of multiple factors to create a coherent representation of the visual environment (Murray et al., 2002). These factors can include the visual environment, nature of the stimuli perceived, individual’s experience, physiological and cognitive processes, context, among many others. In particular, the visual environment and nature of the visual stimuli play a crucial role in shaping visual perception, as they provide the sensory input that our visual system relies on to create a cognitive model of the external world (Simoncelli, 2003).

When considering the content of visual environments, individuals are exposed to a wide range of scenes such as natural, man-made, and computer-generated. Natural scenes have been documented to show characteristic patterns of statistical properties (Torralba and Oliva, 2003). In terms of the power spectrum, natural scenes follow an inverse power law and an anisotropic spectral distribution with lower power at oblique orientations and higher power at horizontal and vertical orientations (Switkes et al., 1978; Burton and Moorhead, 1987; Field, 1987; Tolhurst et al., 1992; van der Schaaf and van Hateren, 1996; Baddeley, 1997; Oliva and Torralba, 2001). This anisotropy has also been described in visual perception through psychophysical experiments (Hansen and Essock, 2004; Mannion et al., 2010; Girshick et al., 2011; Maloney and Clifford, 2015; Raman and Sarkar, 2017), and in the primary visual cortex through neurophysiological studies, which have found more neurons and larger cortical regions tuned to horizontal and vertical orientations compared to oblique ones (De Valois and De Valois, 1988; Chapman and Bonhoeffer, 1998; Coppola et al., 1998; Li et al., 2003; Mannion et al., 2010; Maloney and Clifford, 2015). These studies suggested that such anisotropy might be caused by the nature of the visual environment and demonstrated that the visual system appears to favor horizontal orientation over the vertical.

The anisotropic power distribution in natural scenes and the over-representation of horizontal and vertical orientation-selective neurons in the primary visual cortex have raised questions about whether these neurons are solely responsible for orientation perception or whether they also play a role in processing other visual features, such as size. Groundbreaking studies reported that orientation-selective neurons may also be involved in size perception, which is likely due to the strong correlation between the spatial frequency content of visual stimuli and their orientation (Hubel and Wiesel, 1962; Campbell and Kulikowski, 1966; Gilinsky, 1968; Hubel and Wiesel, 1968; Blakemore and Campbell, 1969; Blakemore and Sutton, 1969; Campbell et al., 1969; Blakemore et al., 1970; De Valois et al., 1982). These investigations have shown that orientation-selective neurons in the primary visual cortex are highly sensitive to specific ranges of spatial frequencies, with different neurons responding preferentially to different spatial frequencies (Hubel and Wiesel, 1962; Hubel and Wiesel, 1968; De Valois et al., 1982; De Valois and De Valois, 1988). Seemingly, these neurons process spatial frequency and orientation in an integrated manner, exhibiting selectivity towards the orientation and dimensions of retinal images, and potentially contributing to the encoding and perception of the size of simple patterns (Blakemore and Campbell, 1969; Blakemore et al., 1970; Blakemore and Nachmias, 1971).

Despite these significant findings, there is still a lack of understanding regarding the influence of multiple factors on the encoding of size information. Considering the importance of the visual input in shaping of perceptual experiences and the intricate neural mechanisms operating within the primary visual cortex, it becomes apparent that our current comprehension of the simple design features and steps of integration in networks underlying size perception remains constrained. To contribute to the understanding of these mechanisms, this study aims to investigate the potential influence of variations in the orientation of the spectral distribution of the visual environment on size perception. By manipulating the predominance of cardinal orientations in a natural scene and examining the effects of adaptation, we seek to shed light on the relationship between spectral distribution biases, orientation-selective neurons, and the encoding of size perception. Our novel approach involves exposing participants to visually biased adaptation stimuli and evaluating their subsequent size perception of simple patterns. To precisely accomplish it, our research relies on manual estimate aperture measurements. This method not only provides a direct assessment of size perception, but also addresses a fundamental problem in visual tasks where stimuli are influenced by identical aftereffects. We hypothesize that adaptation to a spectrally biased natural scene will induce perceptual biases in size estimation, reflecting the influence of orientation-selective neurons and the spectral characteristics of the visual environment.

## 2 Materials and methods

### 2.1 Participants

Twenty healthy adult volunteers (13 women and 7 men) with ages ranging from 25 to 37 years (mean age: 30.68 ± 3.34 years), took part in this experiment. Those volunteers who were left-handed, with known ocular, neurological or musculoskeletal disorders and/or with simultaneous participation in other research studies were excluded from the study. All participants had uncorrected or corrected visual acuities ≥0.0 logMAR. To avoid any optical distortion from spectacles, only emmetropes or contact lens wearers were included.

The study was approved by the Ethics Committee at the Medical Faculty of the Eberhard Karls University Tuebingen and the University Hospital in Tuebingen. The study followed the tenets of the Declaration of Helsinki and signed written informed consent was collected from all participants after having received and understood all study-related details, risks, and benefits.

### 2.2 Visual stimuli

#### 2.2.1 Adaptation stimuli

A natural open-source image sized 1920×1348 pixels was used to generate the adaptation stimuli. To bias the predominance of cardinal orientations (horizontal and vertical) of the original image, an elliptical geometric transformation was applied. On the x-coordinate, each point (x) of the original coordinate system was mapped to a point (x’) in a new coordinate system given by the function (1):


(1) $$f\left(x,y\right)=\tan^{-1}\left(\alpha \cdot \sqrt{\frac{\left(1-{x^{\prime }}^{2}\right)}{x^{\prime }}}\right)\cdot \frac{m_{x}}{\pi }$$

where α is the second radius of the ellipse [relative to the image width ( $m_{x}$ )]. Thus, the effect of the elliptical geometric transformation was defined by α: α = 0.1 (vertical-biased); and α = 4 (horizontal-biased). The y-coordinate was unaltered ( $f\left(x,y\right)=y$ ).

To ensure a constant visual angle of 20° both vertically and horizontally, the image was resized and cropped to a size of 800×800 pixels. To avoid high spatial frequency components caused by boundary effects, image edges were smoothed by means of a Hanning window function (2) (Harris, 1978):


(2) $$w\left(r\right)=\cos^{2}\left(\frac{\pi }{N}\right)\cdot r$$

where r is the radial distance of the pixel position from the center of the image and N is the image width. Applying a Hanning window implied maximum intensity in the image center and progressively reduction towards the periphery.

**Figure 1:**
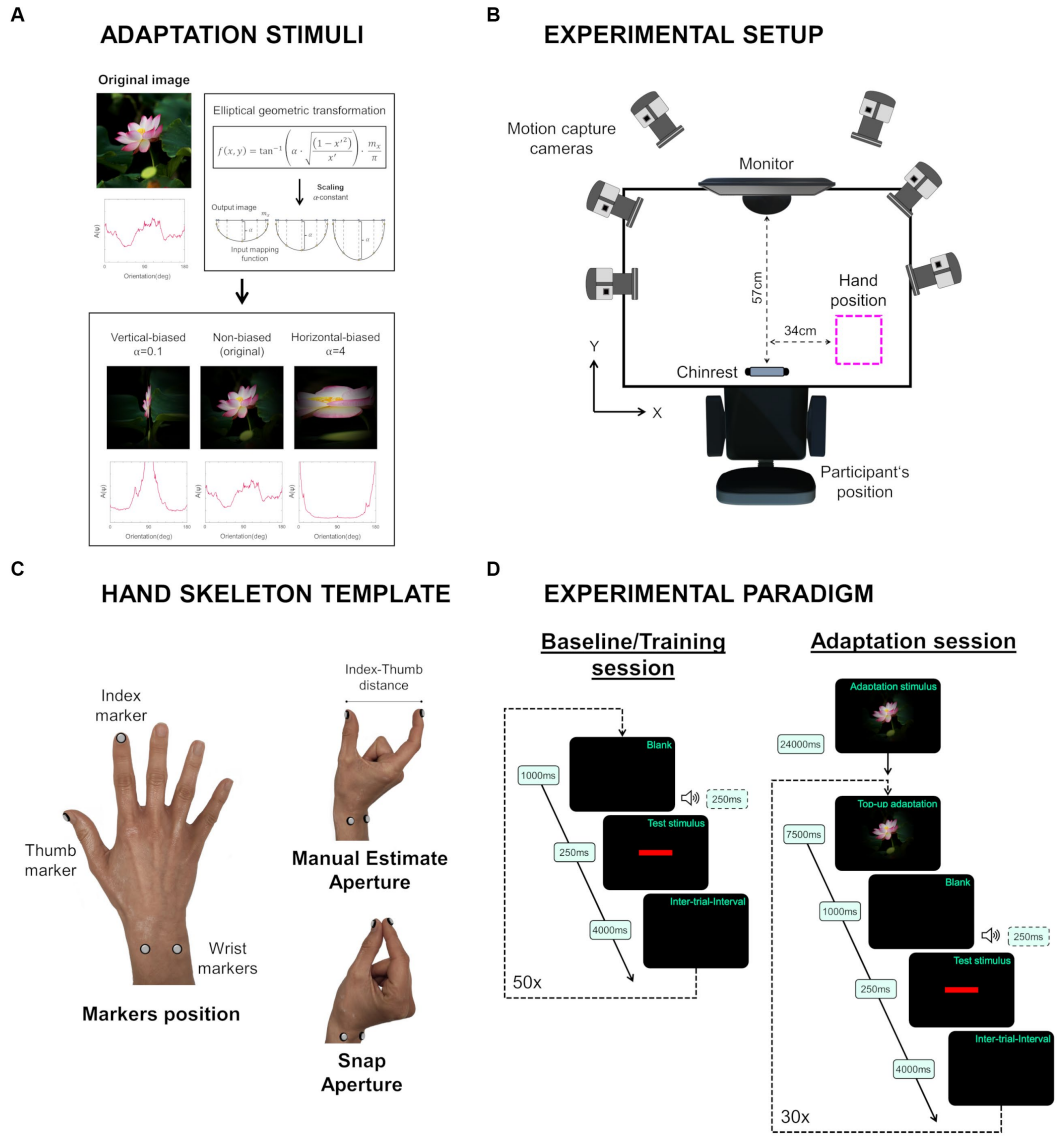
**(A)** Adaptation stimuli. An elliptical geometric transformation was applied to bias the cardinal spectral distribution predominance of the original image. The effect was altered through the α-constant: vertical-biased (α = 0.1); and horizontal-biased (α = 4). To confirm the bias in spectral power orientations, the weighted average of the spectral amplitude [A(ψ)] was analyzed for each α-constant value. **(B)** An overhead view of the experimental setup. **(C)** Schematic representation of the hand skeleton template. Four passive retroreflective markers were attached to the dorsal surface (index nail, thumb nail and wrist). Participants’ responses during size perception were calculated through the distance between the index and thumb markers. To account for digit thickness, real manual estimate aperture was determined by calculating the difference between the index-thumb distance during manual estimate aperture and the index-thumb distance during snap aperture. **(D)** Diagram of the behavioral task.

Figure 1A shows the adaptation stimuli involved in the experimental task. Vertical-biased (α = 0.1) and horizontal-biased (α = 4) stimuli were used as adaptors, while non-biased was used as a control stimulus.

To evaluate the effect of elliptical geometric transformation on the orientation statistics of the original image, the weighted average spectral amplitude distribution A(ψ) was inspected for each orientation [see function (3)]. As noted in Figure 1A, the spectral amplitude A(ψ) of the non-biased stimulus was mainly distributed along the cardinal orientations, vertical and horizontal. In the vertical-biased stimulus, spectral amplitude A(ψ) was biased towards vertical orientation, while in the horizontal-biased stimulus it was biased towards horizontal orientation.


(3) $$A\left(\psi \right)=\frac{\sum_{f}f\cdot A\left(\psi ,f\right)}{m}$$

MATLAB 2020a (The MathWorks, Massachusetts, United States) was used to process and generate adaptation stimuli.

#### 2.2.2 Test stimuli

Ten filled rectangles of 5.3 mm height served as test stimuli to assess the cardinal biased adaptation aftereffects on horizontal size perception. Test stimuli were red and displayed in the center of the screen on a black background. The following ten horizontal sizes were used: 30.0, 33.6, 37.2, 40.8, 44.4, 48.0, 51.6, 55.2, 58.8, and 62.4 mm.

### 2.3 Apparatus and set-up

A diagram of the experimental setup is shown in Figure 1B. Participants were seated comfortably in front of a Full HD 23-inch liquid crystal display monitor (Dell Technologies, Texas, United States) which presented the visual stimuli at a screen resolution of 1920×1080 pixels (0.265 mm pixel pitch) and a refresh rate of 60 Hz. A chin rest was used to stabilize participants’ head position and maintain the viewing distance at 57 cm where the adaptation visual stimulus subtended a visual angle of 20°, horizontally and vertically.

As depicted in Figure 1B, an optoelectronic motion capture system (Vicon, Vicon Motion Systems Ltd., Oxford, UK) composed of a set of six infrared motion capture cameras (Vicon Vero v2.2, Vicon Motion Systems Ltd., Oxford, UK) was used to collect the three-dimensional position of the participants’ right hand. Infrared motion capture cameras were characterized by a 2048×1088 pixel resolution and a maximum frame rate of 330 Hz. For the current study, a sampling rate of 100 Hz was set. A 26-Port PoE+ Switch (DGS-1026MP, D-Link Corporation, Taipei, Taiwan) served to power the optical cameras and connect them to the host computer (Dell Technologies, Texas, United States) using Ethernet cabling.

Nexus software (Nexus 2.10, Vicon Motion Systems Ltd., Oxford, UK) enabled us to manage the different system components, as well as the camera and calibration processes, acquisition, storage, visualization, and processing of all the three-dimensional data associated with each experimental trial.

The optical motion capture system employed in this study is classified as passive, since it uses retro-reflective markers which send the infrared light back to the sensors. Then, Nexus software relies on the collected optical information to reconstruct and calculate the position of the markers in three-dimensional space. In the current study, four passive retroreflective markers were used to monitor the three-dimensional trajectory of the participants’ right hand. As shown in Figure 1C, these markers were placed on the dorsal surface of the hand: one marker on the thumb nail, one on the index fingernail, and two on the wrist (ulnar and radius heads). All markers were spherical in shape, 6 mm in diameter, made of a hard pearl material, and glued onto a soft base. Double-sided fixing tape was used to attach the markers on the participants’ hand.

Participants’ right-hand position was bounded by a rectangular-shaped area created with adhesive strips, at 34 cm from the midline of the body (Figure 1B). As a result, the right hand was kept out of the participants’ field of view during the entire experiment.

The psychophysical paradigm was designed and generated using Python 3.7 (Van Rossum and Drake, 2009) and its psychophysics cross-platform package, PsychoPy (Peirce, 2007). An external laptop (Dell Technologies, Texas, United States) was used to execute the custom Python scripts. The speaker of the external laptop was used to provide auditory feedback during the experimental task. The generated auditory signal was a sine wave with a frequency of 2,500 Hz.

Ambient room illumination was kept steady during all measurements. Black cardboard screens were installed on the lateral sides of the table to avoid unwanted lights entering the capture volume and participants’ field of view.

### 2.4 Procedure

#### 2.4.1 System calibration

After setting up the system (positioning, orientation, and adjustment of camera parameters) and before each participant’s measurements, both the system calibration and the capture volume establishment were conducted.

The optical camera calibration was supported by the so-called Active Wand, a T-shaped electronic motion capture calibration device (Active Wand, Vicon Motion Systems Ltd., Oxford, UK), which contains 5 pairs of LEDs. The calibration device was waved through the intended capture volume until the cameras registered 1,000 frames of valid wand data. Once the optical calibration was completed, the global coordinate system was established. Using three retroreflective markers, the volume capture origin, and therefore the capture center and its orientation (x, y, z) were determined.

#### 2.4.2 Behavioral task

The experimental paradigm comprised four sessions: a baseline/training session and three adaptation sessions categorized as non-biased, horizontal-biased, and vertical-biased.

In the baseline/training session, participants completed 50 trials separated by a 4,000 ms inter-trial interval. Each trial began with a 1,000 ms blank interval coupled with a 250 ms auditory signal, after which the test stimulus was displayed at the center of the screen for a duration of 250 ms. Following the test stimulus presentation, participants were instructed to manually estimate the horizontal size of the test stimulus (Figure 1D). This session allowed participants to become familiar with the estimation task and enabled us to assess any potential impact of the non-biased condition on size perception.

The three adaptation sessions followed a similar experimental flow. Each session started with an initial adaptation of 24,000 ms, during which the adaptation stimulus was continuously displayed. Subsequently, each trial included a 7,500 ms top-up adaptation using the same adaptation stimulus as in the initial adaptation. After the top-up adaptation, a 1,000 ms blank interval coupled with a 250 ms auditory signal preceded the presentation of the test stimulus for 250 ms. Participants were then instructed to manually report the horizontal size of the test stimulus. Each adaptation session consisted of 30 trials with a 4,000 ms inter-trial interval (Figure 1D). The adaptation stimulus used in each session determined its category: non-biased, horizontal-biased, or vertical-biased. The order of the adaptation sessions was randomized, and participants had a 10-min break between sessions. The entire experimental process was completed in approximately 90 min.

Before the experiment, all participants received a detailed briefing on the experimental procedure, including instructions on how to accurately estimate the horizontal size of the test stimulus. For the manual estimation, participants were instructed to lift their right hand approximately 5 to 10 cm above the surface plane, rotate their wrist clockwise, and indicate the size of the test stimulus by extending their index finger and thumb. Natural right-hand movements and accurate size estimations were emphasized. When viewing the stimuli, participants were instructed to observe them naturally, without any restrictions on blinking or gaze. Additionally, participants were reminded to avoid fixating on their right hand while making size estimations.

The experiment was conducted in a darkened room under binocular viewing conditions. Right-hand dominance was assessed using the Flinders Handedness survey (Nicholls et al., 2013) prior to the beginning of the experiment.

### 2.5 Data processing and statistical analysis

Offline frame-by-frame data processing analysis followed three steps: (1) reconstruct and label, (2) gap-filling, and (3) data filtering. Once all trials were labeled and gap filled, marker coordinates (x, y, z) were filtered using a fourth-order Butterworth low pass filter with a cutoff frequency of 6 Hz. Custom MATLAB algorithms (The MathWorks, Massachusetts, United States) were created to compute three-dimensional marker velocities and subsequently calculate the distance between index and thumb markers (known as manual estimate aperture, see Figure 1C). As in our previous work (Sanz Diez et al., 2022), to estimate participants’ horizontal size perception, manual estimate aperture values were solely considered in those time periods where index and thumb marker velocities were below 5 mm/s. Within those intervals, the manual estimate aperture was identified as the maximum index-thumb distance.

To avoid digit thickness, the real manual estimate aperture (in mm), referred to as the distance between the inner surfaces of the index finger and thumb, was calculated. For that, each participant was given a 5-s measurement in which they had to bring the fingerprints of their index finger and thumb together (as in a finger snap, see Figure 1C). The distance between the index and thumb markers during this 5-s measurement was named “snap aperture.” The real manual estimate aperture was then calculated as the difference between the index-thumb distance from each frame and the mean “snap aperture”.

Given that the non-biased condition contained both horizontal and vertical components, it served as a control in our experiment. As a result, we assessed the size estimation aftereffects by comparing the non-biased condition with the vertical- and horizontal-biased conditions. The manual estimate aperture shift (Δ manual estimate aperture) between non-biased and vertical- or horizontal-biased adaptation conditions was calculated as an aftereffect metric. The differences in manual estimate aperture values between the non-biased condition with the vertical- and horizontal-biased conditions served to compute the percentage changes (Percentage Change = ((Estimated value in vertical- or horizontal-biased condition – Estimated value in non-biased condition)/Estimated value in non-biased condition) * 100). Additionally, real and estimated size values were used to calculate the percent accuracy of size estimation as follows: ((Real value – Estimated value)/Real value) × 100.

Statistical analysis was performed under MATLAB R2020a statistical toolbox (The MathWorks, Massachusetts, United States). The data distribution was examined for normality using the Kolmogorov–Smirnov test. Given the non-normal distribution, the data underwent appropriate statistical analyses, which included Mann–Whitney U-test, Kruskal-Wallis test and Wilcoxon signed rank test. Furthermore, Spearman’s rank correlation and *post hoc* analyses using the Dunn’s approach were performed. The significance level was set at *p* values < 0.05. Results are provided as mean ± standard error of the mean.

## 3 Results

**Figure 2:**
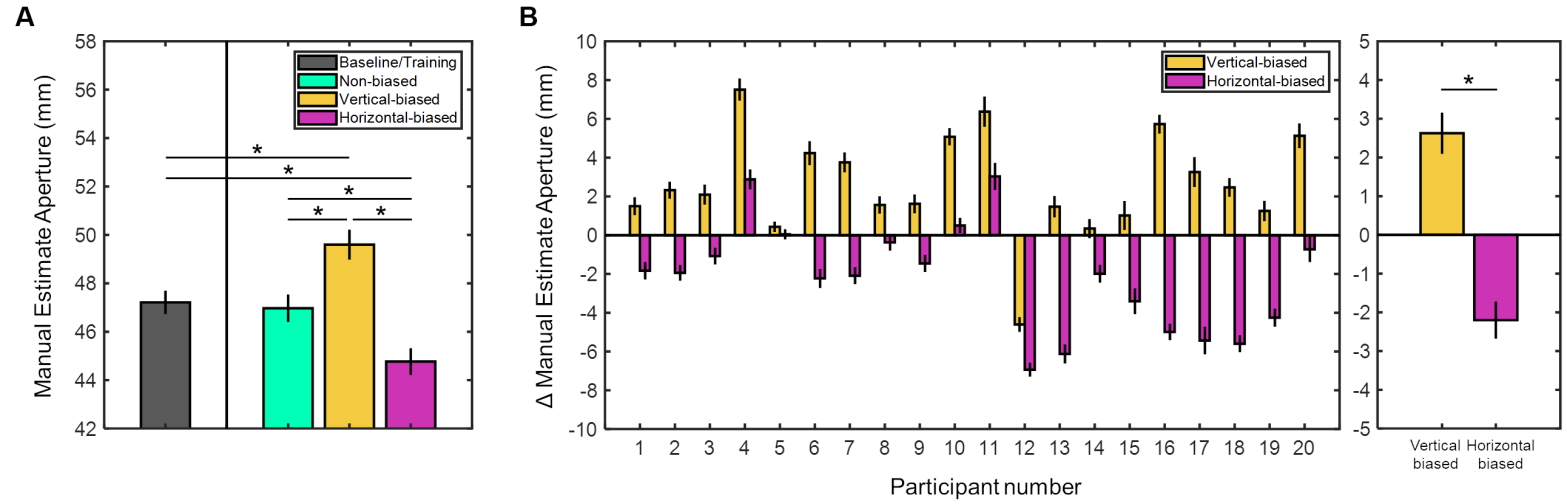
**(A)** Mean manual estimate aperture values (in millimeters) for all experimental sessions: baseline/training, vertical-biased, non-biased, horizontal-biased. **(B)** Individual manual estimate aperture shift values (in millimeters) for two adaptation conditions: vertical-biased (gold bars) and horizontal-biased (magenta bars). Considering non-biased as control, vertical-biased showed a positive shift while horizontal-biased exhibited a negative shift. The mean values derived from the manual estimate aperture shifts are shown in the rightmost bar graph. Asterisks indicate *p*-values < 0.05. Error bars denote the standard error of the mean.

To investigate the adaptation aftereffects on size perception, we assessed the manual estimate aperture values for all participants across the four experimental sessions. Figure 2A shows the mean manual estimate aperture values for all participants in all sessions. On average, manual estimate aperture values were 47.21 ± 0.48 mm (baseline/training), 46.97 ± 0.57 mm (non-biased), 49.60 ± 0.62 mm (vertical-biased), and 44.77 ± 0.55 mm (horizontal-biased). Values differed significantly among sessions (χ2(2) = 27.85, *p* < 0.001, Kruskal-Wallis test). *Post hoc* analyses indicated that average manual estimate aperture values were significantly higher in the vertical-biased condition than in the other three adaptation conditions: baseline/training (*p* = 0.005), non-biased (*p* = 0.005), and horizontal-biased (*p* < 0.001). Conversely, horizontal-biased condition presented significantly lower manual estimate aperture values relative to baseline/training, non-biased, and vertical-biased (*p* ≤ 0.01 for all). Non-biased and baseline/training conditions showed similar manual estimate aperture responses (*p* = 0.76).

Figure 2B illustrates the individual and averaged adaptation aftereffects on size perception for vertical-biased and horizontal-biased conditions. A manual estimation shift towards positive values was observed after the vertical-biased adaptation (2.63 ± 0.53 mm), while a negative shift was detected after the horizontal-biased adaptation (−2.20 ± 0.48 mm). Manual estimate aperture was significantly shifted from non-biased results in both adaptation conditions, vertical- and horizontal-biased (*p* < 0.001, *z* = −3.65; and *p* < 0.001, *z* = 3.34; respectively; Wilcoxon signed rank test). Adaptation aftereffects on size perception were significantly diverse in vertical- and horizontal-adaptation conditions (*p* < 0.001, *z* = −4.95; Mann–Whitney U-test). These results revealed notable variations in how participants perceived the horizontal sizes after vertical- and horizontal-biased adaptation conditions. Compared to the non-biased condition, participants perceived horizontally larger sizes after vertical-biased adaptation, whereas smaller sizes after horizontal-biased adaptation. Moreover, as observed when contrasting non-biased and baseline/training results, horizontal size perception was not affected by previously displaying the non-biased visual adaptation stimulus.

**Figure 3:**
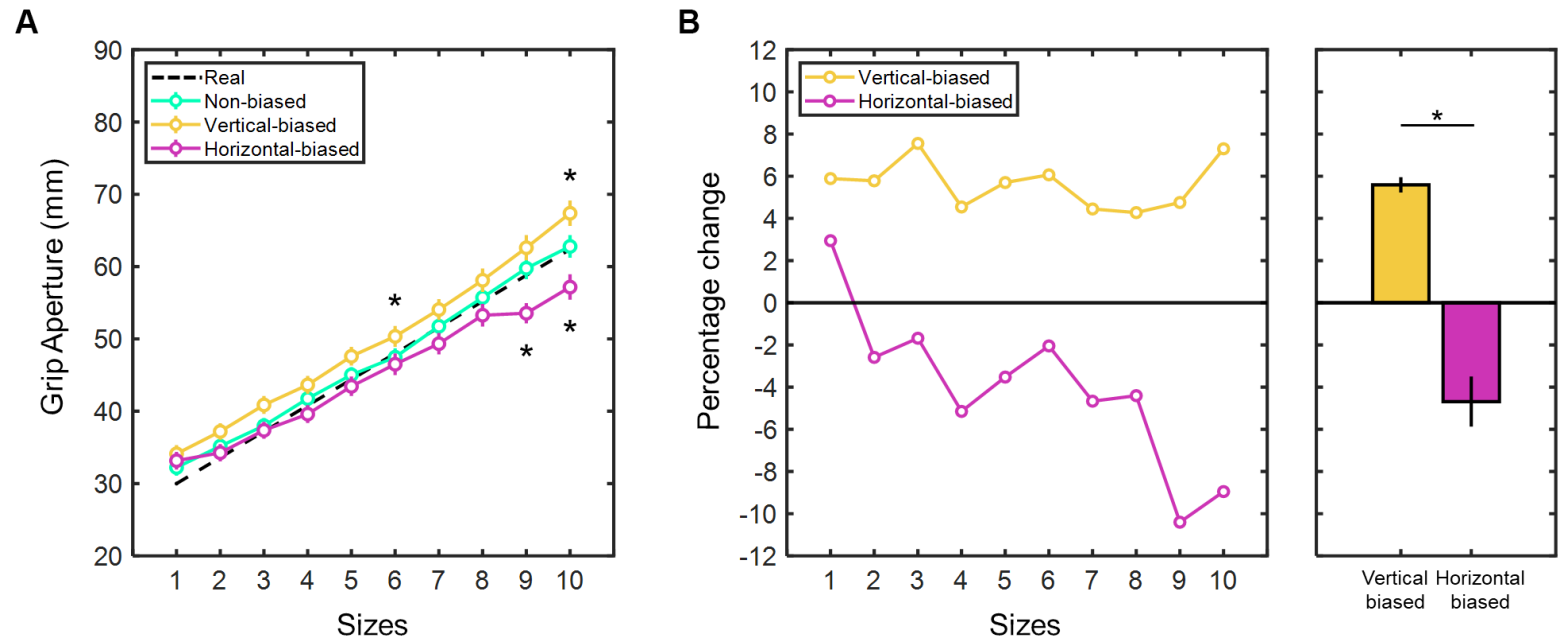
**(A)** Mean manual estimate aperture values (in millimeters) for each test stimulus size following the three adaptation conditions: vertical-biased (gold); non-biased (green); and horizontal-biased (magenta). Black dotted line indicates the real sizes of the test stimulus (sizes in ascending order, from 1 to 10: 30.0, 33.6, 37.2, 40.8, 44.4, 48.0, 51.6, 55.2, 58.8, and 62.4 mm). Error bars represent the standard error of the mean. Asterisks indicate significant deviation (*p* < 0.05) from non-biased. **(B)** Percentage change in the perceived horizontal size of the test stimuli after vertical-biased (gold) and horizontal-biased (magenta) conditions, relative to non-biased condition and as a function of test stimulus sizes. Average percent change values are shown in the rightmost bar graph. Error bars indicate the standard error of the mean. Asterisks imply *p*-values less than 0.05. Test stimulus sizes in ascending order, from 1 to 10: 30.0, 33.6, 37.2, 40.8, 44.4, 48.0, 51.6, 55.2, 58.8, and 62.4 mm.

Additionally, the mean manual estimate aperture values were determined for each test stimulus size across the three adaptation conditions (Figure 3A). Consistently, the larger the real test stimulus sizes, the higher the manual estimate aperture values (χ2(9) = 304.52, *p* < 0.001 for non-biased; χ2(9) = 275.66, *p* < 0.001 for vertical-biased; and χ2(9) = 210.91, *p* < 0.001 for horizontal-biased; Kruskal-Wallis test), thus indicating a consistent pattern of size estimation responses. Spearman’s rank correlation confirmed a significant positive association between manual estimate aperture responses and real test stimulus sizes in the three conditions (*r* > 0.99, *p* < 0.001). Therefore, participants were able to make clear distinctions among the different sizes used for the test stimuli. Comparisons between the manual estimate aperture values of the non-biased condition and the vertical- and horizontal-biased conditions revealed contrasting trends in the percentage change (Figure 3B). Following the vertical-biased condition, observers overestimated the horizontal size of the test stimulus by 5.59 ± 0.37%. Conversely, after the horizontal-biased condition, observers underestimated the horizontal size by 4.69 ± 1.19%.

**Figure 4:**
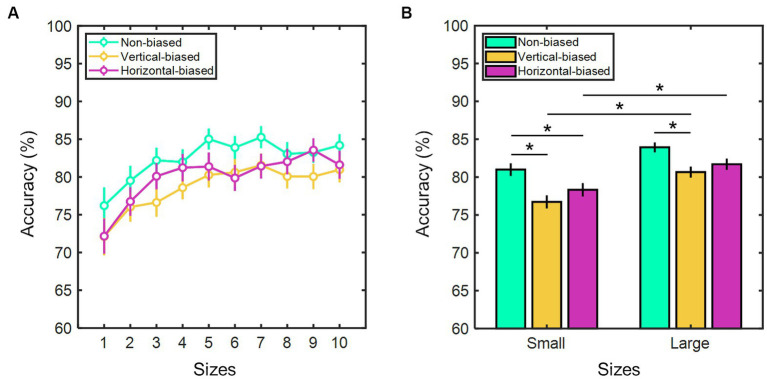
**(A)** Percent accuracy of size estimation based on test stimulus sizes (from 1 to 10: 30.0, 33.6, 37.2, 40.8, 44.4, 48.0, 51.6, 55.2, 58.8, and 62.4 mm) and **(B)** as a function of two groups of test stimulus sizes (small: sizes ranging from 30.0 to 44.4 mm; and large: sizes ranging from 48.0 to 62.4 mm) for the three adaptation conditions: vertical-biased (gold); non-biased (green); and horizontal-biased (magenta). Error bars indicate the standard error of the mean. Asterisks imply *p*-values less than 0.05.

Considering the relationship between perceived sizes and real sizes of the test stimuli, we examined adaptation aftereffects on size perception accuracy. High accuracy rates were observed in all four conditions. On average, 81.36 ± 0.43% for baseline/training, 82.46 ± 0.54% for non-biased, 78.69 ± 0.57% for vertical-biased, and 80.01 ± 0.59% for horizontal-biased. A Kruskal-Wallis test determined that accuracy rates varied significantly among conditions (χ2(3) = 27.97, *p* < 0.001). Non-biased adaptation showed significantly higher accuracies than vertical-biased (*p* < 0.001) and horizontal-biased conditions (*p* < 0.001). Baseline/training and non-biased had comparable accuracies (*p* = 0.12). In all three adaptation conditions, a Spearman rank correlation analysis showed a significant positive correlation between accuracy rates and real test stimulus sizes (*r* = 0.74, *p* = 0.02 for non-biased; *r* = 0.84, *p* = 0.003 for vertical-biased; and *r* = 0.79, *p* = 0.01 for horizontal-biased). This suggests that as test stimulus size increases, participant’s accuracy rates also tend to increase (Figure 4A). In fact, as shown in Figure 4B, accuracy rates were notably lower for the group comprising smaller test stimulus sizes, ranging from 30.0 to 44.4 mm, in comparison to the group consisting of larger test stimulus sizes, ranging from 48.0 to 62.4 mm. In the non-biased condition, the group associated with smaller sizes exhibited an average accuracy of 80.98 ± 0.84%, whereas the group linked to larger sizes demonstrated a mean accuracy of 83.93 ± 0.66%. When considering the vertical-biased and horizontal-biased conditions, the group of smaller sizes yielded mean accuracy rates of 76.72 ± 0.87% and 78.32 ± 0.89%, respectively, while the group of larger sizes yielded 80.65 ± 0.73% and 81.70 ± 0.76% (*z* = −2.83, *p* = 0.005 for vertical-biased; *z* = −2.48, *p* = 0.01 for horizontal-biased). Notably, in both the vertical-biased and horizontal-biased, the accuracy rates for the group of larger sizes were significantly higher by approximately 3.5% (*z* = −2.83, *p* = 0.005 for vertical biased; *z* = −2.48, *p* = 0.01 for horizontal biased). Conversely, the non-biased condition did not reveal any significant accuracy differences between the smaller and larger size groups (z = −1.86, *p* = 0.06). Among all three conditions, accuracy rates did not differ statistically for each of the test stimulus sizes (for all conditions at each of the sizes: χ2(2) ≥ 1.78, *p* ≥ 0.09, Kruskal-Wallis test).

## 4 Discussion

The present study aimed to examine aftereffects on size perception after short exposure to a natural scene with a biased spectral amplitude distribution. Specifically, we assessed the perception of the horizontal size of a rectangle following short-term adaptation to a natural scene containing either a natural bias (non-biased: with a predominant orientation at 0° and 90°) or an induced bias (vertical-biased: with a predominant orientation at 90°; and horizontal-biased: with a predominant orientation at 0°) in the spectral amplitude distribution. To quantify perceptual shifts, we monitored the three-dimensional trajectories of hand movements and computed the manual estimation aperture values, as a behavioral metric, during size estimations. This approach allowed us to accurately evaluate whether exposure to a natural scene with concrete spectral orientation biases can change size perception in a controlled psychophysical experiment.

The results demonstrated that participants showed significant changes in size perception responses after exposure to vertical-biased and horizontal-biased stimuli, indicating adaptation aftereffects in both conditions. Compared to the non-biased condition, participants perceived horizontally larger sizes after the vertical-biased adaptation condition whereas smaller sizes after the horizontal-biased adaptation condition. Specifically, after viewing the vertical-biased stimulus observers overestimated the horizontal size of the rectangle by ~6%. After viewing the horizontal-biased stimulus the horizontal size of the rectangle was underestimated by ~5%. That is, adaptation aftereffects on size perception revealed a bidirectional pattern in both adaptation conditions. Our findings are consistent with those described by Frome et al. (1979). They assessed size perception of a solid luminous rectangle after viewing a vertically or horizontally oriented sinusoidal grating. In a series of trials, following the adaptation period, participants had to complete two alternative tasks: to manually adjust the vertical dimension of the rectangle to make it appear square, or to verbally identify a square in a series of randomly presented rectangles. In line with our outcomes, their investigation demonstrated that size perception was influenced along the dimension orthogonal to the spatial frequency orientation. That is, after adaptation to vertically oriented sinusoidal gratings participants perceived the rectangle as wider, whereas, after adaptation to horizontally oriented sinusoidal gratings participants perceived the rectangle as taller, indeed, narrower (Frome et al., 1979). Interestingly, they found a size aftereffect of 6%, which is very close to what we have observed in our results, a 5–6% shift.

Although with a different methodological approach, similar findings were also reported by Taya and Miura (2007). In their study, participants were asked to estimate the width and depth of reference stimuli consisting of projected squares with an elliptical cylindrical appearance. These squares were oriented vertically and depicted using texture or luminance gradients. Specifically, observers were required to adjust the width of an outlined rectangle to match the width of the reference stimulus (square with an elliptic cylindrical appearance). The authors noted that, for both texture and luminance stimuli, the width of the reference stimulus was perceived as narrower than the outlined rectangle. That is, as observed in our study, the vertically oriented elliptical cylinder generated an overestimation of the horizontal perception of size. This effect was dependent on the cylindrical depth perception: the greater the depth, the stronger the horizontal shrinkage effect. In fact, larger effects were reported for those reference stimuli with higher percentages of change ratio in line widths and luminance (30% and 16%, respectively). For both texture and luminance stimuli, they obtained an average ratio of 0.95 (Taya and Miura, 2007). Considering the 44-mm width of the reference stimulus, a mean ratio of 0.95 would imply that observers perceived the outlined rectangle as ~2.3 mm wider than the reference stimulus. This would interestingly translate into a size aftereffect of ~5.3%, which is very similar to that reported by Frome et al. (1979) and to that found in the current study under the vertical-biased adaptation condition. A vertical shrinkage effect was also reported when the cylindrical texture stimulus was rotated 90°. Using this approach, the researchers observed that participants perceived the heights of the horizontally oriented elliptical cylinders to be smaller than those of the outlined rectangles. In particular, the greater the depth, the greater the magnitude of shrinkage in perceived height. A mean maximum ratio of 0.91 was computed, which means a ~ 9.9% size aftereffect in height (Taya and Miura, 2007). After suggesting some contributing factors to the shrinkage effect (such as pictorial cues, visibility of the stimulus edge, number of stimulus lines, and uniformity of stimulus line width) the researchers argued that the participants’ visual system might have been biased by the cylindrical and three-dimensional appearance of the stimuli, i.e., by the apparent depth. They consider that, from a perceptual point of view, given the apparent depth, judging the width or height of the cylinder based on its edges can be a difficult task. This is because the edges are defined by the points where the cylinder tangent plane is perpendicular to the frontal plane. Therefore, from a front view position and considering the apparent depth, the visual system would not be accurate enough to identify the exact points of the physical edges thus generating narrower perceptual size responses (Taya and Miura, 2007).

**Figure 5:**
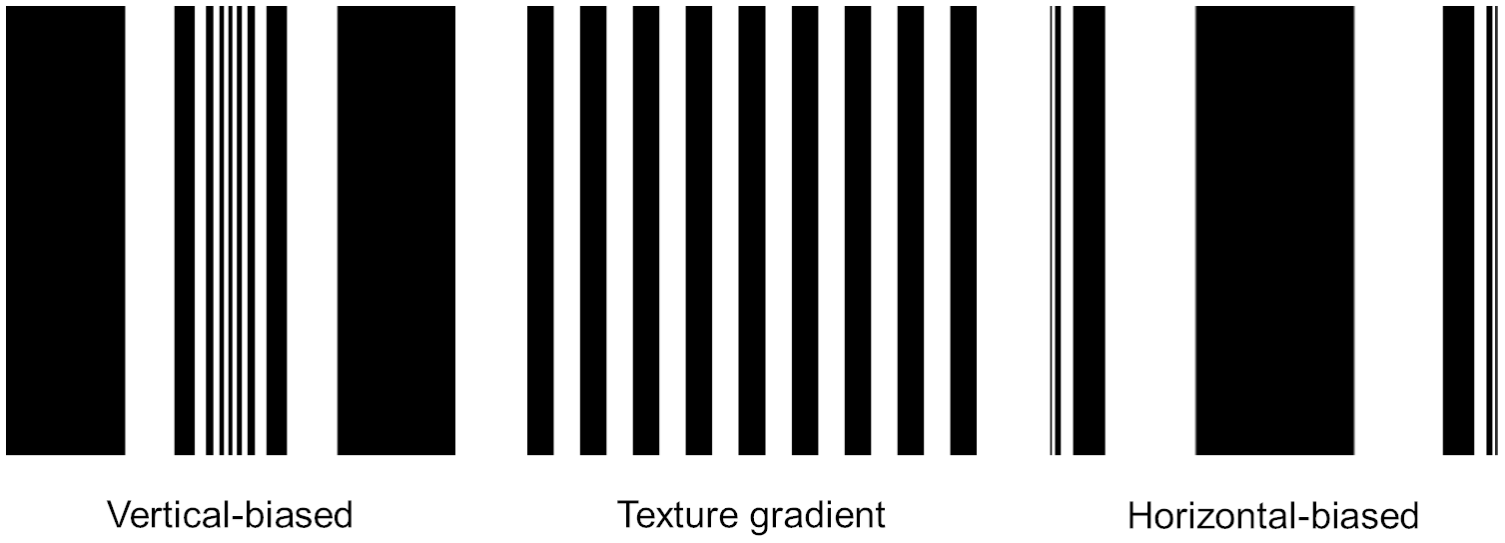
Implementation of the elliptical geometric transformation [see function (1)] on a texture gradient composed of nine black and eight white lines. The geometric transformation was applied considering the two values of α-constant employed in the current study: α = 0.1 (vertical-biased) and α = 4 (horizontal-biased). The effect observed in the horizontal-biased condition is analogous to the effect generated by the line width modulation reported in Taya and Miura (2007).

Perhaps, the same assumption could be extended to our results. For example, by applying solely the elliptical geometric transformation used in our study [see function (1)] to a texture gradient formed by nine black and eight white lines, as reported by Taya and Miura (2007), we obtain the elliptical modulation represented in Figure 5 for both vertical-biased and horizontal-biased conditions. As can be seen, the effect generated in the horizontal-biased condition is very similar to the texture stimulus reported in their study. However, despite the possible similarity in cylindrical appearance, it is important to consider several aspects of our study. Firstly, we applied a Hanning window that reduced edge effects. Secondly, as an adaptation stimulus, we employed a natural image which was characterized by their complexity and variety of visual features. Finally, the experimental task in our study was to estimate the horizontal size of a rectangle which was independent of the adaptation stimulus. We, therefore, believe that the size aftereffects found in our study could have been triggered by factors other than those reported by Taya and Miura (2007).

Our findings might be interpreted as evidence for contrastive processes that operate in the visual system. Contrastive processes serve to intensify the differences between adaptor stimulus and subsequent inputs (Storrs and Arnold, 2017) and have already been reported in various visual properties such as orientation (Gibson and Radner, 1937; Mitchell and Muir, 1976), shape (Badcock et al., 2014; Storrs and Arnold, 2017), or even more complex spatial properties (Zhao et al., 2011; Ross et al., 2014; Storrs and Arnold, 2015). For instance, despite methodological differences (we keep the aspect ratio of adaptation stimuli constant, and our adaptor and test stimuli are independent), the contrastive aftereffect trend observed in aspect ratio adaptation studies closely resembles the size aftereffects mentioned in our study. Aspect ratio adaptation modulates shape perception: after adapting to an ellipse that is elongated horizontally, a circle may seem to be elongated vertically; whereas, after adapting to an ellipse that is elongated vertically, a circle may seem to be elongated horizontally (Regan and Hamstra, 1992; Suzuki and Cavanagh, 1998; Suzuki, 2005; Badcock et al., 2014; Storrs and Arnold, 2017). In our study, the bias in the spectral distribution magnified the difference between the adaptation environment and subsequent visual inputs, and therefore, the adaptation mechanisms updated the visual context and altered the perception of the spatial properties of the consecutive test stimuli. Such adaptation mechanisms could be linked to multi-channel encoding schemes that process the orientation, spatial frequency, and size of retinal images. Indeed, multi-channel encoding predicts locally repulsive aftereffects that are relative to the features of the adaptation environment and subsequent stimuli (Suzuki, 2005; Storrs and Arnold, 2017). The size aftereffects encountered here could result from the multi-channel encoding of spectral distribution by those neurons in the visual cortex that are simultaneously tuned to orientation and spatial frequency.

Building upon prior research, our findings offer additional support for the hypothesis that spatial frequency mechanisms may contribute to the regulation of size information encoding. Under the current experimental approach, after the exposure to the non-biased adaptation stimulus, participants perceived the test stimuli to be closer in size to their actual dimensions. This was corroborated by comparing the perceptual responses of the baseline/training and non-biased conditions, and further evidenced by the percentage accuracy results seen in Figure 4. These findings underscore that the size aftereffect we noted cannot be solely attributed to a figural aftereffect arising from scene elongation in either horizontal or vertical directions, but rather points towards a more intricate interplay of factors. Apparently, adaptation to a visual environment dominated by both horizontal and vertical spatial frequency components failed to bias the perception of horizontal size towards a specific direction. A balanced spatial distribution kept the perceived size of the test stimulus unaffected. However, when the spectral amplitude distribution was biased towards a concrete orientation, size perception was altered. Adaptation to a given spectral distribution orientation would depress the sensitivity of those neural mechanisms that respond best to that amplitude orientation. Following adaptation, the perceived horizontal size of the test stimulus would be shifted correspondingly as a result of these neural elements becoming less responsive to the orientation of the spectral distribution of the adaptation stimulus. For example, when the participant’s visual system was exposed to a vertical-biased stimulus, neurons that were selective for vertical orientation became less responsive due to their decreased sensitivity to stimuli with vertical components. This may cause a shift in the balance of activity between neurons selective for vertical and horizontal orientation. As a result, when participants viewed the test stimulus after adaptation, the rectangle appeared wider, maybe due to the relatively higher activity of neurons selective for horizontal orientation compared to those selective for vertical orientation. Similarly, when the visual system adapted to horizontal-biased stimulus, neurons that were selective for horizontal orientation became less responsive due to their decreased sensitivity to stimuli with horizontal components. Consequently, following adaptation, the test stimulus seemed narrower. In both scenarios, local repulsion mechanisms would shift size perception away from the most adapted orientation spectrum.

In summary, visual perception is shaped by a complex interplay of factors, including the visual environment, the nature of the stimuli being perceived, the individual’s previous experience, and the physiological and cognitive processes that underlie perception. Our research offers further evidence supporting the significance of spatial frequency in the perception of size. The results suggest that the visual system is sensitive to the statistical regularities of the visual environment and adapts accordingly. These findings may have significant implications for our comprehension of the processes and representations employed by the visual system to handle visual information. Nevertheless, it is important to note that our study was limited using a single natural scene as an adaptor. This approach, while providing initial insights, may not fully capture the complexity and variability inherent in natural scenes. The potential influence of different spectral amplitude distributions on size perception remains an open question. In future research, we plan to address this limitation by incorporating a diverse array of natural images into our experimental paradigm. This will allow us to examine the robustness of our findings and further elucidate the intricate interplay between natural scene statistics and perceptual biases. We believe that this expanded scope will significantly enhance our understanding of size perception in naturalistic contexts.

## Data availability statement

The raw data supporting the conclusions of this article will be made available by the authors, without undue reservation.

## Ethics statement

The study was approved by the Ethics Committee at the Medical Faculty of the Eberhard Karls University Tuebingen and the University Hospital in Tuebingen. The study followed the tenets of the Declaration of Helsinki and signed written informed consent was collected from all participants after having received and understood all study-related details, risks, and benefits.

## Author contributions

PSD and SG conceived and planned the study. PSD performed data collection and data analysis. PSD wrote the first draft of the manuscript. PSD, SG, AB, PF, and SW conducted the subsequent revisions and provided technical and intellectual aspects for the study. PF and SW served as project manager for the study. All authors contributed to the article and approved the submitted version.
